# Genomic regions and pathways associated with gastrointestinal parasites resistance in Santa Inês breed adapted to tropical climate

**DOI:** 10.1186/s40104-017-0190-4

**Published:** 2017-09-04

**Authors:** Mariana Piatto Berton, Rafael Medeiros de Oliveira Silva, Elisa Peripolli, Nedenia Bonvino Stafuzza, Jesús Fernández Martin, Maria Saura Álvarez, Beatriz Villanueva Gavinã, Miguel Angel Toro, Georgget Banchero, Priscila Silva Oliveira, Joanir Pereira Eler, Fernando Baldi, José Bento Sterman Ferraz

**Affiliations:** 1 Departamento de Zootecnia, Faculdade de Ciências Agrárias e Veterinárias, Universidade Estadual Paulista, Via de acesso Prof. Paulo Donato Castellane, s/no, Jaboticabal, SP CEP 14884-900 Brazil; 2 Instituto Nacional de Investigación y Tecnología Agraria y Alimentaria INIA, Crta. de la Coruña, km 7,5 -, 28040 Madrid, Spain; 3 Departamento de Producción Agraria, School of Agricultural, Food and Byosystems Engineering, Universisdad Politécnica de Madrid, Campus Ciudad Universitaria Avda. Complutense 3 - Avda. Puerta Hierro, 28040 Madrid, Spain; 4 Instituto Nacional de Investigación Agropecuária (INIA), Ruta 50 Km. 12, Colonia, Uruguay; 5 Faculdade de Zootecnia e Engenharia de Alimentos, Nucleo de Apoio à Pesquisa em Melhoramento Animal, Biotecnologia e Transgenia, Universidade de São Paulo, Rua Duque de Caxias Norte, 225, Pirassununga, SP CEP 13635-900 Brazil

**Keywords:** Gwas, Linkage disequilibrium, Parasites resistance, Santa Inês breed

## Abstract

**Background:**

The aim of this study was to estimate variance components and to identify genomic regions and pathways associated with resistance to gastrointestinal parasites, particularly *Haemonchus contortus,* in a breed of sheep adapted to tropical climate. Phenotypes evaluations were performed to verify resistance to gastrointestinal parasites, and were divided into two categories: i) farm phenotypes, assessing body condition score (BCS), degree of anemia assessed by the famacha chart (FAM), fur score (FS) and feces consistency (FC); and ii) lab phenotypes, comprising blood analyses for hematocrit (HCT), white blood cell count (WBC), red blood cell count (RBC), hemoglobin (HGB), platelets (PLT) and transformed (log_10_) egg per gram of feces (EPG_log_). A total of 576 animals were genotyped with the Ovine SNP12k BeadChip (Illumina, Inc.), that contains 12,785 bialleleic SNP markers. The variance components were estimated using a single trait model by single step genomic BLUP procedure.

**Results:**

The overall linkage disequilibrium (LD) mean between pairs of markers measured by *r* ^2^ was 0.23. The overall LD mean between markers considering windows up to 10 Mb was 0.07. The mean LD between adjacent SNPs across autosomes ranged from 0.02 to 0.10. Heritability estimates were low for EPG_log_ (0.11), moderate for RBC (0.18), PLT (0.17) HCT (0.20), HGB (0.16) and WBC (0.22), and high for FAM (0.35). A total of 22, 21, 23, 20, 26, 25 and 23 windows for EPG_log_ for FAM, WBC, RBC, PLT, HCT and HGB traits were identified, respectively. Among the associated windows, 10 were shown to be common to HCT and HGB traits on OAR1, OAR2, OAR3, OAR5, OAR8 and OAR15.

**Conclusion:**

The traits indicating gastrointestinal parasites resistance presented an adequate genetic variability to respond to selection in Santa Inês breed, and it is expected a higher genetic gain for FAM trait when compared to the others. The level of LD estimated for markers separated by less than 1 Mb indicated that the Ovine SNP12k BeadChip might be a suitable tool for identifying genomic regions associated with traits related to gastrointestinal parasite resistance. Several candidate genes related to immune system development and activation, inflammatory response, regulation of lymphocytes and leukocytes proliferation were found. These genes may help in the selection of animals with higher resistance to parasites.

**Electronic supplementary material:**

The online version of this article (doi:10.1186/s40104-017-0190-4) contains supplementary material, which is available to authorized users.

## Background

Small ruminants, like sheep (*Ovis aries*) and goats (*Capra aegagrus hircus*) were the first animals domesticated by human for food supply, being the most important group of ruminants raised in temperate and tropical regions [1] . Sheep are multi-purpose animals, raised for meat, milk, wool, hides and skins, having a huge socioeconomic importance worldwide. However, there are several productive drawbacks associated with gastrointestinal parasites infection in small ruminants, since it represents the type of disease with the highest impact on animal health and productivity [2]. Thus, the losses caused by gastrointestinal parasites and the costs due to excessive use of anthelmintic drugs are a struggling problem that restricts the sheep production in many regions of the world.

The main gastrointestinal nematodes of small ruminants belong to the *Trichostrongylidae* family. These parasites occur in the gastrointestinal tract and are seen as the main obstacle in the sheep industry, since they lead to significant economic losses due to high mortality and productivity losses. Within the *Trichostrongylidae* family, the *Haemonchus* genera has great pathogenic action and is the most prevalent parasite affecting small ruminants, mainly in tropical regions, where environmental conditions are characterized by high temperature and humidity, and abundant rainfall during summer [3–6].

To reduce the economic losses caused by nematode infections there are several management alternatives to minimize the damage, such as raise breeds or animals that are more resistant to these infections [5]. In this regard, Santa Inês, an American hair breed, showed higher resistance to gastrointestinal nematode infections when compared to European sheep breeds [5, 7–9]. Several studies have shown that selection for gastrointestinal parasite resistance is possible in sheep, since genetic progress in research and commercial herds [10–16].

The identification of genetic markers associated with gastrointestinal parasites resistance could increase the genetic response for this trait by marker-assisted selection [15]. Furthermore, the identification of genomic regions associated with resistance or susceptibility to gastrointestinal parasites will help to deeper understand the biological and physiological processes underlying this trait [17]. Several studies reported genetic markers associated with gastrointestinal parasites resistance close to the Major Histocompatibility Complex (MHC) [18–20] and *IFN-gama* genes [18–22]. Recently, genome-wide association studies have identified genetic variants for gastrointestinal parasite resistance in some sheep breeds [23–26]. The identification of genomic regions that play a role in gastrointestinal parasite resistance may become an important tool to improve the resistance of Santa Inês or other sheep breeds. Therefore, the aim of this study was to estimate variance components and to identify genomic regions and pathways associated with resistance to gastrointestinal parasites in a Santa Inês population adapted to tropical conditions.

## Methods

### Data

The phenotypic records were collected from 700 naturally infected animals of Santa Inês breed belonging to four flocks located in the Minas Gerais and São Paulo southeast states and Sergipe northeast state of Brazil. The samples collection and phenotypes evaluations were performed from October to November of 2011. Phenotypes evaluations were achieved to verify resistance to gastrointestinal parasites, and were divided into two categories: i) farm phenotypes, assessing body condition score (BCS), degree of anemia assessed by the famacha card (FAM), fur score (FS) and feces consistency (FC); and ii) lab phenotypes, comprising blood analyses for hematocrit (HCT), white blood cell count (WBC), red blood cell count (RBC), hemoglobin (HGB), platelets (PLT) and the egg counts per gram of feces (EPG_log_). The EPG values were transformed to log_10_ (*n* + 1) = EPG_log_ to meet the basic requirements of normality and homogeneity in an attempt to stabilize the variance prior to analyses, where *n* is the number of EPG_log_ per sample.

Blood samples were collected by puncture of the jugular vein using vacuum tubes with anticoagulant EDTA and clot activator. Subsequently, samples were submitted to the Veterinary hematology analyzer Sysmex PocH-100iV Diff to perform a complete blood cell count for HCT, WBC, RBC, HGB and PLT. Fecal samples used for EPG evaluation were taken directly from the rectum of the animals and sent to the laboratory for analyses. The EPG count was assessed using the modified McMaster technique [27] and the parasites’ genera were identified using the morphometric key by Van Wyk et al. [28]. The feces were classified through visual inspection according to its consistency appearance (FC) developed by Gordon et al. [29] and modified by the authors considering three out of the original five-value scale: 1 for normal stool, 2 for pasty stool, and 3 for diarrheal feces.

The BCS was assessed after evaluating the prominences of the spinous and transverse bones of the spine, fat coverage, and muscle development between the last rib and the ileum wing, as described by Russel et al. [30]. Coloration of the ocular mucosa was measured by trained people by observing the medial part of the lower conjunctiva and comparing it with the famacha chart (FAM; FAMACHA© System) considering a five-value scale: 1 for red robust, 2 for rosy red, 3 for pink, 4 for pale pink, and 5 for white [31]. The descriptive statistics for the analyzed traits are presented in Table 1.

**Table 1:** Descriptive statistics for eggs per gram of feces (EPG_log_), red blood cells (RBC), famacha (FAM), platelet (PLT), white blood cells (WBC), hematocrit (HCT), and hemoglobin (HGB)

Trait	*n*	Mean	SD^a^	CV^b^, %	Min	Max
EPG_log_	517	2.57	0.53	20.6	1.71	4.04
RBC, 10^6^/μL	513	9.97	2.12	21.2	4.39	19.25
FAM	518	3.08	0.97	31.16	1.0	5.0
PLT, 10^3^/μL	514	383.8	241.6	62.9	8.0	2101.0
WBC, 10^3^/μL	514	10.96	3.73	34.0	3.60	44.40
HCT, %	514	29.05	6.79	23.4	10.90	58.00
HGB, g/dL	514	8.43	1.94	23.0	3.30	17.30

^a^SD: Standard deviation; ^b^CV: Coefficient of variation

### Genotyping of animals

The extraction of the genomic DNA from each animal was performed from blood samples collected with EDTA. For this, red blood cells were lysed using 1 mL of whole blood and 500 μL of lysis solution (0.32 mol/L Sucrose, 12 mmol/L Tris-HCl pH 7.5, 5 mmol/L MgCl_2_, 1% Triton X-100) followed by centrifugation at 13,000 r/min. The supernatant was discarded to reach the leucocytes. The pellets were slowly washed adding 1 mL of ultrapure water and then the microtube was poured out of the water and held for a few minutes on absorbent paper to completely dry the pellet. This washing step was repeated approximately three times until a clean white pellet was obtained. The white pellet was then kept frozen (−20 °C) until sending to the DEOXI laboratory in Araçatuba-SP, where the DNA was completely extracted. The DNA of each sample was quantified and the degree of purity (ratio of optical densities between 260 and 280 nm) (Table 1) was evaluated. After these processes, the samples were stored at −20 °C until genotyping was performed.

A total of 576 animals were genotyped with the Ovine SNP12k BeadChip (Illumina, Inc.), that contained 12,785 biallelic SNP markers. Quality control consisted of excluding markers with unknown genomic position, located on sex chromosomes, monomorphic, with minor allele frequency (MAF) lower than 0.05, call rate lower than 90%, and with excess heterozygosity. After quality control, there were 11,602 SNPs and 574 samples left for analyses.

### Quantitative genetic analyses

The variance components were estimated using a single animal trait model by single step genomic BLUP (ssGBLUP) procedure, under Bayesian inference [32]. For all studied traits the fixed effects considered in the model were contemporary groups (farm and management group), month of sample collection, sex, covariable body condition (linear effect), and age at the collection (linear and quadratic effect). All phenotypes were tested for data consistency and contemporary groups with less than three animals and records out of three standard deviations from the contemporary group mean were discarded, remaining 500 animals with phenotypes records. The ssGBLUP is a modified version of the animal model (BLUP) with additive relationship matrix ***A*** ^***−1***^ replaced by ***H*** ^***−1***^ [33]:

$$ {H}^{-\mathbf{1}}={A}^{-\mathbf{1}}+\left[\begin{array}{cc}\hfill \mathbf{0}\hfill & \hfill \mathbf{0}\hfill \\ {}\hfill \mathbf{0}\hfill & \hfill {G}^{-\mathbf{1}}-{A}_{\mathbf{22}}^{-\mathbf{1}}\hfill \end{array}\right] $$

where ***A***_***2*2**_ is a numerator relationship matrix for genotyped animals and ***G*** is the genomic relationship matrix created as described by [34]:

$$ G={ZDZ}^{\hbox{'}}q $$

where ***Z*** is the gene matrix containing allele frequency adjustment; ***D*** is the matrix that have the SNP weight (initially ***D*** *=* ***I***); and, *q* is a weighting/standardization factor. According to [35], such factors can be obtained by ensuring that the ***G*** average diagonal is next to ***A***_***22***_. The pedigree file has a total of 1196 animals and the last three generations of animals with records were considered. A linear model was used to analyze HCT, WBC, RBC, HGB, PLT, and EPG_log_. The model can be represented by the following matrix equation:

$$ y=\mathrm{X}\upbeta + Za+e $$

where ***y*** is the observations vector; ***β*** is the vector of fixed effects; *a* is the additive direct vector; ***X*** is the incidence matrix; ***Z*** is the incidence genetic random effects additive direct matrix (the ***β*** vector associated with the y vector); ***e*** is the residual effect vector. The priori distributions of vectors ***y***, ***a*** and ***e*** were given by:

$$ {\displaystyle \begin{array}{c}\hfill y\sim MVN\left( X\beta + Za\right)\hfill \\ {}\hfill a\mid G\sim MVN\left( 0,H\otimes G\right)\hfill \\ {}\hfill e\mid R\sim \mathrm{MVN}\left( 0,I\otimes R\right)\hfill \end{array}} $$

where ***H*** is the relationship coefficients matrix among animals obtained from the single-step analyses (single-step); ***R*** is the residual variance matrix; ***I*** is the identity matrix; ***G*** is genetic additive variance matrix and ⨂ is the Kronecker product. An inverted chi-square distribution was used for the prior values of the direct and residual genetic variances. A uniform distribution was used a priori for the fixed effects.

A threshold model was applied to analyze the FAM trait. The scores of FAM for each individual *i*, were defined by *Ui* in the underlying scale *yi =* (1) *t*_*0*_ *< Ui < t*_*1*_ *;* (2) *t*_*1*_ *< Ui < t*_*2*_ *;* (3) *t*_*2*_ *< Ui < t*_*3*_ *; (4) t*_*3*_ *< Ui < t*_*4*_ *; i* = 1, … *n*, where *n* is the number of observations; *t*_*1*_ to *t*_*4*_ were the threshold values; and *U* is the unobservable continuous variable, in underlying scale, limited between two unobservable thresholds. After specifying the thresholds *t*_*0*_ to *t*_*4*_ it is necessary to adjust one of the thresholds (from *t*_*1*_ to *t*_*4*_) into an arbitrary constant. In the present study, it was assumed that *t*_*1*_ = 0, in such a way that the vector of estimable thresholds was defined as *t = t*_*2*_ *, t*_*3*_ *, t*_*4*_. After defining the model parameters, the link between categorical and continuous scales could be established based on the contribution of the probability of an observation that fitted the first category, which is proportional to:

$$ P\left( yv=0|t,0\operatorname{}\right)=P\left( Uv<t|t,0\operatorname{}\right)=\Phi \left[\left(t-{w}^{'} vo\right)/{\sigma}_e\right] $$

where *yv* is the response variable for the *vth* observation; *t* is the threshold value arbitrarily assigned as the true value is unobservable; *Uv* is the value of the underlying variable for the *vth* observation; (*ϕ*) is the cumulative distribution function of a standard normal variable; and *w’v* is the scale of the incidence matrix that linked θ to the *vth* observation. As the observations are conditionally independent, given θ, the likelihood function is defined by the product of contributions of each record.

The analyses were performed using the GIBBS2F90 and THRGIBBSF90 programs [33, 36]. The a posteriori variance component estimates were obtained using the POSTGIBBSF90 program [36]. The analysis consisted of a single chain of 500,000 cycles discarding the first 20,000 cycles, taking a sample at every 100 iterations. Thus, 48,000 samples were used to obtain the parameters. The data convergence was verified with the graphical evaluation of sampled values versus interactions according to the criteria proposed by several authors [37–39], using the Bayesian Output Analysis (BOA) of the R 2.9.0 software [40].

### Linkage disequilibrium estimation

The linkage disequilibrium (LD) between two SNPs was evaluated using *r* ^2^ as follows:

$$ {r}^2={\frac{\left( freq.{AB}^{\ast } freq. ab- freq.{Ab}^{\ast } freq. aB\right)}{\left( freq.{A}^{\ast } freq.{a}^{\ast } freq.{B}^{\ast } freq.b\right)}}^2=\frac{(D)^2}{\left( freq.{A}^{\ast } freq.{a}^{\ast } freq.{B}^{\ast } freq.b\right)} $$

where,

$$ D= freq. AB- freq.{A}^{\ast } freq.B $$

And

$$ {D}^{\hbox{'}}=\left\{\begin{array}{c}\hfill \frac{D}{\min \left( freq.{A}^{\ast } freq.b, freq.{a}^{\ast } freq.B\right)}\kern0.24em if\kern1em D<0\hfill \\ {}\hfill \frac{D}{\min \left( freq.{A}^{\ast } freq.B, freq.{a}^{\ast } freq.b\right)}\kern0.24em if\kern1em D<0\hfill \end{array}\right\} $$

where *freq.A, freq.a, freq.B* and *freq.b* are the frequencies of alleles A, a, B and b, respectively, and *freq.AB, freq.ab*, *freq.aB* and *freq.Ab* are the frequencies of haplotypes Ab, ab, aB and Ab in the population, respectively. The expected frequency of haplotype AB (*freq.AB*) is calculated as the product between *freq.A* and *freq.B*. The *r* ^2^ takes values close to zero when the alleles A and B segregate independently. The *freq.AB* higher or lower than the expected value indicates that these two loci in particular tend to segregate together and are in LD, with a maximum value for *r* ^2^ of one.

### Principal component analysis

The principal component analyses (PCAs) were obtained from the genomic relationship matrix through the preGS90 program. The preGS90 is an interface program to process genomic information for the BLUPF90 family [36]. Efficient methods for the creation of the genomic relationship matrix, relationship based on genomic data, and their inverses are described by [41]. The PCAs applied to genotype data can be used to calculate principal components (PCs) that explain differences among individual samples in the genetic data. The top PCs are viewed as continuous axes of variation that reflect genetic variation due to ancestry in the sample. Individuals with similar values for a particular principal component will have a similar ancestry for that axes.

### Genome-wide association analysis

The genome-wide association analysis for each trait was performed using the single-step GWAS (ssGWAS) methodology [42]. The same linear animal model for HCT, WBC, RBC, HGB, PLT and EPG_log_, and the threshold model for FAM used to estimate the variance components were applied. The effects were decomposed in genotyped (a_g_) and non-genotyped (a_n_) animals as describe by [42], considering the effect of genotyped animals as:

$$ {\mathrm{a}}_g=Z\mathrm{u}, $$

where ***Z*** is a matrix that relates genotypes of each locus and *u* is a vector of marker effects, and the variance of animal effects was assumed as:

$$ \operatorname{var}\left({\mathrm{a}}_g\right)=\operatorname{var}\left(\mathrm{Zu}\right)={\mathrm{ZDZ}}^{\hbox{'}}{\sigma}_u^2={G}^{\ast }{\sigma}_{\mathrm{a}}^2 $$

where ***D*** is a diagonal matrix of weights for variances of markers (***D*** *=* ***I*** for GBLUP), ${\sigma}_{\mathrm{u}}^2$ is the genetic additive variance captured by each SNP marker when no weights are present, and ***G*** * is the weighted genomic relationship matrix. The ratio of covariance of genetic effects (*a*_*g*_) and SNPs (*u*) is:

$$ \operatorname{var}\left[\begin{array}{c}\hfill {a}_t\hfill \\ {}\hfill \mathrm{u}\hfill \end{array}\right]=\left[\begin{array}{cc}\hfill {\mathrm{ZD}\mathrm{Z}}^{\hbox{'}}\hfill & \hfill {\mathrm{ZD}}^{\hbox{'}}\hfill \\ {}\hfill {\mathrm{DZ}}^{\hbox{'}}\hfill & \hfill D\hfill \end{array}\right]{\sigma}_{\mathrm{u}}^2, $$

Sequentially:

$$ {\mathbf{G}}^{\ast}\kern0.5em =\frac{\operatorname{var}\left({\mathrm{a}}_{\mathrm{g}}\right)}{\sigma_{\mathrm{a}}^2}=\frac{\operatorname{var}\left(\mathrm{Zu}\right)}{\sigma_{\mathrm{a}}^2}={\mathrm{ZDZ}}^{\hbox{'}}\lambda $$

where λ is a variance ratio or a normalizing constant. According to [34],

$$ \lambda =\frac{\sigma_{\mathrm{a}}^2}{\sigma_{\mathrm{a}}^2}=\frac{1}{\sum_{\mathrm{i}=1}^{\mathrm{M}}2{\mathrm{p}}_{\mathrm{i}}\left(1-{\mathrm{p}}_{\mathrm{i}}\right),} $$

where *M* is the number of SNP and *p*_*i*_ is the allele frequency of the second allele of the *i* ^*th*^ SNP. According to [43], the markers effect can be described by:

$$ \widehat{\mathrm{u}}=\frac{\upsigma_u^2}{\upsigma_u^2}\mathrm{DZ}\hbox{'}\mathbf{G}{\ast}^{-1}{\widehat{\mathrm{a}}}_{\mathrm{g}}={\mathrm{DZ}}^{\hbox{'}}{\left[{\mathrm{ZDZ}}^{\hbox{'}}\right]}^{-1}{\widehat{\mathrm{a}}}_{{\mathrm{g}}^{\hbox{'}}} $$

The estimated SNP effects can be used to estimate the variance of each individual SNP effect [44] and apply a different weighting for each marker, such as:

$$ {\hat{\upsigma}}_{\hat{\mathrm{u}},\mathrm{i}}^2={\hat{\mathrm{u}}}_i^22{\mathrm{p}}_{\mathrm{i}}\left(1-{\mathrm{p}}_{\mathrm{i}}\right) $$

The following iterative process described by [42] was used considering ***D*** to estimate the SNP effects:

1. ***D = I***;
2. To calculate the matrix G = ZDZ^'^q**G** = **ZDZ** ^′^q

3. To calculate GEBVs for all animals in data set using ssGBLUP;

4. To calculate the SNP effect: $\widehat{\mathrm{u}}={\uplambda \mathrm{DZ}}^{'}{\mathrm{G}}^{\ast -1}{\widehat{\mathrm{a}}}_g$

5. To calculate the variance of each SNP:${\mathrm{d}}_{\mathrm{i}}={\widehat{\mathrm{u}}}_{\mathrm{i}}^22{\mathrm{p}}_{\mathrm{i}}\left(1-{\mathrm{p}}_{\mathrm{i}}\right)$, where *I* is the *i-th* marker;

6. To normalize the values of SNPs to keep constant the additive genetic variance;

7. Exit, or loop to step 2.

The markers effects were obtained by two iterations from step 2 to 7. The percentage of genetic variance explained by *i-th* region was calculated as described by [45].

$$ \frac{\mathrm{Var}\left({\mathrm{a}}_{\mathrm{i}}\right)}{\upsigma_{\mathrm{a}}^2}\times 100=\frac{\mathrm{Var}\left({\sum}_{\mathrm{j}=1}^{10}{\mathrm{Z}}_{\mathrm{j}}{\widehat{\mathrm{u}}}_{\mathrm{j}}\right)}{\upsigma_{\mathrm{a}}^2}\times 100 $$

where *a*_*i*_ is genetic value of the i-th region that consists of 10 consecutive SNPs, ${\upsigma}_a^2$ is the total genetic variance, *Z*_*j*_ *Z*_*j*_is vector of gene content of the *j-th* SNP for all individual, and ${\widehat{\mathrm{u}}}_j$ ${\widehat{u}}_j$ is marker effect of the *j-th* within the *i-th* region. Considering a length of the windows size not exceeding 3 to 4 Mb, the results were presented as the proportion of genetic additive variance explained by windows of 10 consecutive SNPs.

### Search for genes

The chromosome regions that explained more than 1.0% of the additive genetic variance were selected to explore and determine possible quantitative trait loci (QTL). The Map Viewer tool of ovine (*Ovis aries*) genome was used for identification of genes, available at “National Center for Biotechnology Information” (NCBI - http://www.ncbi.nlm.nih.gov) database, using the bank references of HuRef assembly, CHM1 1.0, CRA TCAGchr7v2 and Ensembl Genome Browser (http://www.ensembl.org/index.html). The classification of the genes according to its biological function, identification of metabolic pathways and gene enrichment was performed by DAVID tool v6.8 [46] and GeneCards (http://www.genecards.org/). Gene ontology (GO; *P* < 0.01) and Kyoto Encyclopedia of Genes and Genomes (KEGG; *P* < 0.05) pathways were identified by DAVID tool v6.8 [46]. All annotated genes in the *Ovis aries* genome were used as background.

## Results and discussion

### Linkage disequilibrium and population structure

**Fig. 1:**
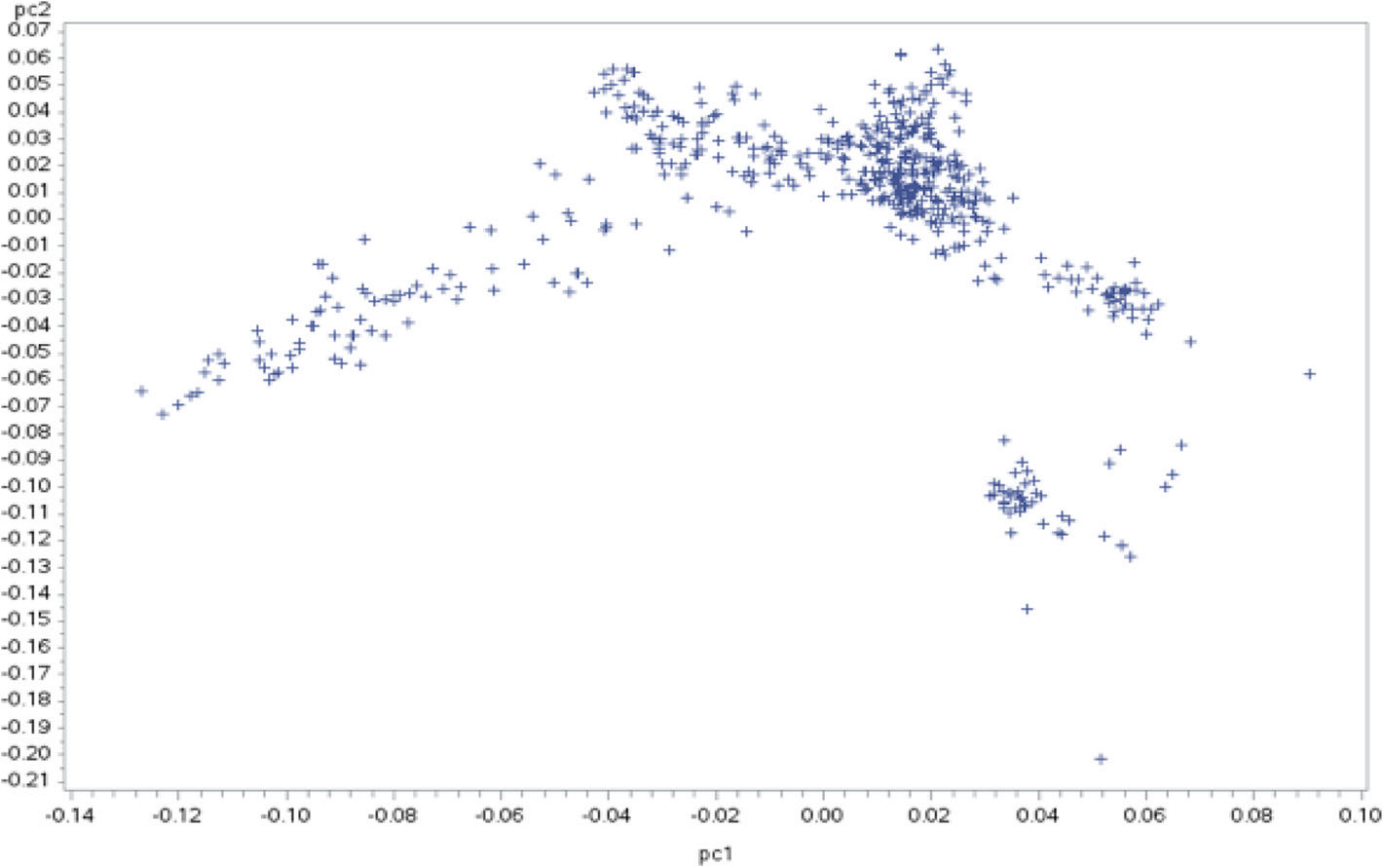
First two principal components (pc1: 3.16%;pc2:15%) of the genomic relationship matrix of genotyped animals

The Fig. 1 represents the first two principals (PC1 and PC2) component analysis (PC) based on the genomic relationship matrix. Despite the animals being originated from flocks located in different regions of Brazil, it is important to note that there was no subpopulation structure in this population.

**Fig. 2:**
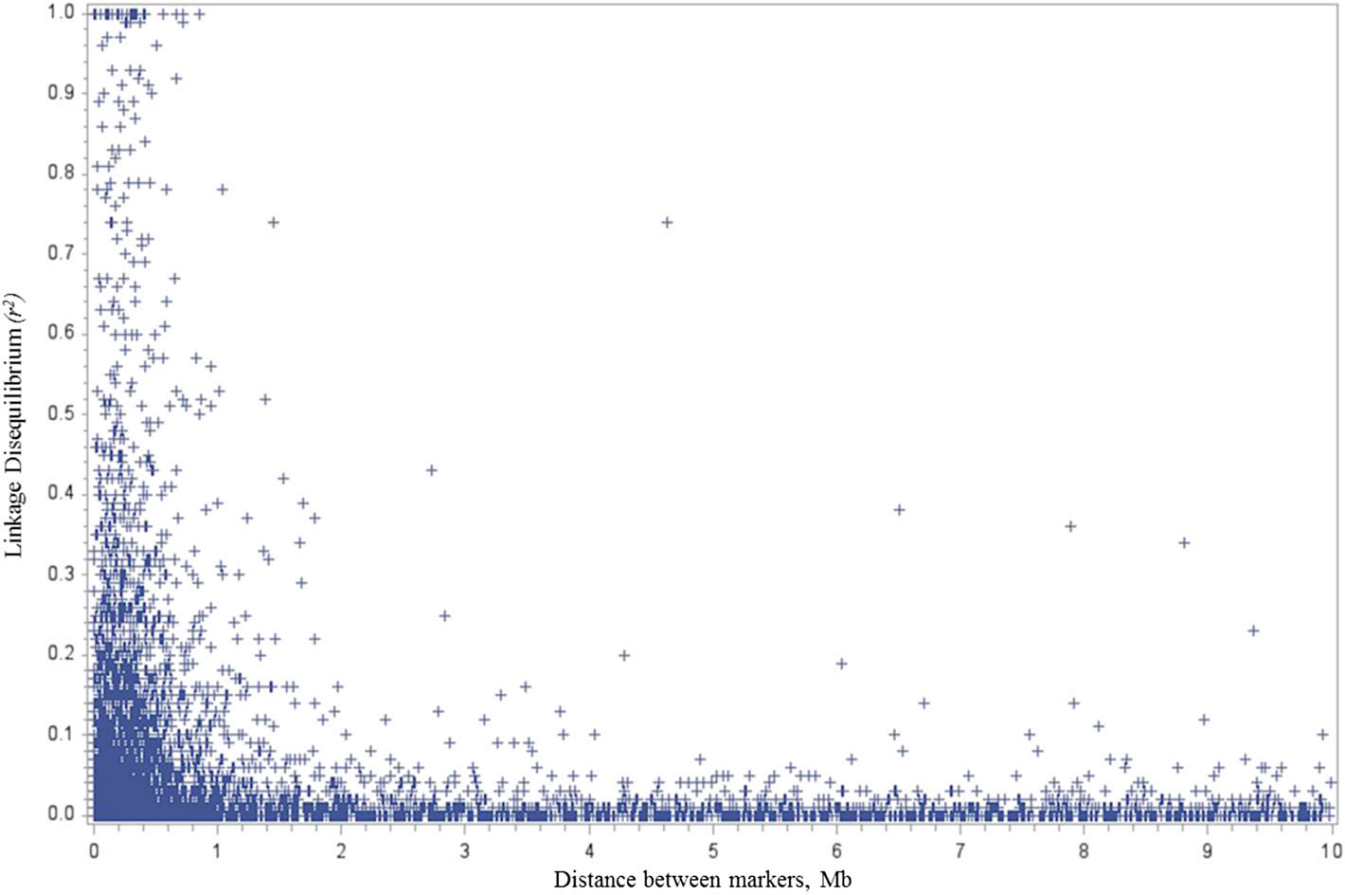
Linkage disquilibrium (*r* ^2^) between markers considering windows up yo 10 Mb

The predicted values of LD vs. linkage distance between genetic markers were presented in Fig. 2. On the basis of this figure, it is possible to state that when considering a distance between markers lower than 1 Mb, the level of LD indicates that the Ovine SNP12k BeadChip may be a suitable tool for identifying genomic regions associated with traits related to gastrointestinal parasites resistance. Most tightly linked SNP pairs have the highest *r* ^2^ and average *r* ^2^ rapidly decreases as linkage distance increases (Fig. 2). The overall LD mean between markers considering windows up to 10 Mb was 0.07. The LD mean between adjacent SNPs across autosomes ranged from 0.02 to 0.10 (Table 2). Several authors have study the pattern of LD between markers in the genome of various sheep breeds [47–52]. It is difficult to compare the level of LD obtained in different studies since different sample sizes, LD measures, marker types, marker densities and historical population demographics [51] were used. However, the level of LD obtained in the present study was similar to those reported in previous studies for wool sheep breeds using the Ovine SNP50 BeadChip [50–52].

**Table 2:** Summary of SNP markers analyzed and average linkage disequilibrium (LD; *r* ^2^) between synthetic adjacent markers obtained for each autosome (OAR)

OAR	*n*	Mean LD	SD^a^ LD	Mean Distance, Mb	SD^a^ Distance, Mb
1	707	0.09	0.17	0.75	1.5
2	683	0.09	0.15	1.01	1.9
3	222	0.04	0.04	2.60	3.0
4	297	0.05	0.09	2.64	3.1
5	369	0.10	0.19	0.72	1.5
6	112	0.03	0.10	2.7	2.5
7	79	0.02	0.04	3.4	2.8
8	288	0.06	0.13	1.9	2.8
9	313	0.07	0.14	1.3	2.3
10	108	0.04	0.10	3.7	2.8
11	171	0.06	0.11	1.5	2.5
12	224	0.06	0.12	1.9	2.7
13	228	0.07	0.14	2.1	2.6
14	131	0.07	0.14	3.0	3.1
15	253	0.08	0.16	1.5	2.5
16	257	0.08	0.16	1.7	2.4
17	152	0.07	0.16	2.6	3.1
18	190	0.07	0.12	1.7	2.5
19	87	0.03	0.09	2.6	2.9
20	137	0.03	0.10	3.4	2.9
21	168	0.06	0.16	2.1	2.8
22	137	0.03	0.06	2.2	2.7
23	201	0.08	0.12	0.9	1.7
24	125	0.09	0.17	1.6	2.1
25	84	0.02	0.11	3.2	2.6
26	175	0.07	0.13	0.93	1.7

^a^SD: Standard deviation

### Genetic parameters estimates

The descriptive statistics for FAM indicates an incidence of animals with some degree of anemia (mainly levels 4 and 5) and animals that are not affected (levels 1 and 2) (Table 1). In the present study, animals were free of other sources that lead to anemia, i.e. fluke or minerals deficiencies such as copper, so, it is possible to asseverate that the presence of anemia in these animals was probably due to the prevalence of a *Haemonchus* population and the susceptibility or not of these ewes to infection. Similarly, the descriptive statistics for EPG_log_ indicates an infection by gastrointestinal parasites (Table 1). Most of the gastrointestinal parasites belonging to the *Trichostrongylidae* family have similar shape and eggs size, and unless the EPG_log_ is accompanied by a larvae cultivar, the eggs can belong to any specie. Moreover, the larva 4 and 5 of *Haemonchus* develops in the wall of the stomach with two particularities: (i) they feed with blood and (ii) do not lay eggs, which make FAM more precise than EPG_log_ to predict infection by *Hemonchus contortus* in all damaging stages.

The variance components and heritability estimates for EPG_log_, RBC, FAM, WBC, PLT, HCT, and HGB were described in Table 3. The parameter estimates convergence was verified by inspection of trace-plots [37, 38] in which the convergence diagnosis indicates the convergence of the chain. Thus, the burn-in period used was considered enough to achieve the convergence of the estimates of all parameters. The marginal posterior distributions of heritability estimates for the traits showed accurate values, tending to a normal distribution, since the mean and the median were similar (Table 3). Symmetric distributions of central tendency statistics were an indicative that the analysis is reliable.

**Table 3:** Estimates of additive genetic variance (Va), residual variance (Vr), and heritability (*h* ^2^) for log_10_ of eggs per gram of feces (EPG_log_), red blood cells (RBC), famacha (FAM), platelets (PLT), white blood cells (WBC), hematocrit (HCT), and hemoglobin (HGB)

Trait	Va	Vr	*h* ^*2*^	SD^a^	Median	HPDi	HPDs
EPG_log_	0.13	1.03	0.11	0.08	0.09	0.001	0.28
RBC	0.45	2.01	0.18	0.10	0.17	0.004	0.37
FAM	0.03	0.06	0.35	0.11	0.35	0.14	0.58
PLT	6.14	28.77	0.17	0.09	0.17	0.02	0.35
WBC	2.71	9.27	0.22	0.10	0.22	0.03	0.41
HCT	4.93	19.04	0.20	0.11	0.19	0.001	0.41
HGB	0.29	1.44	0.16	0.10	0.15	0.001	0.36

^a^SD: heritability standard deviation; HPDi: lower limit of credibility interval (95%) for heritability posterior distribution; HPDs: upper limit of credibility interval (95%) for heritability posterior distribution

Heritability estimates were low for EPG_log_, moderate for RBC, PLT, HCT, HGB and WBC, and high for FAM. Studies involving Santa Inês breed are scarce in the literature and most of them refers to genetic parameters for traits related to parasites resistance performed in wool sheep breeds. Bisset et al. [53] reported heritability estimate for resistance and resilience of sheep to *Haemonchus contortus* in a South African Merino flock and observed high heritability for EPG, FAM and HCT, with values ranging from 0.47 to 0.55. Contrasting with our results, moderate heritability estimates for EPG among different studies were reported. Bishop et al. [54] studying Texel lambs observed a weighted average heritability of 0.26 and 0.38 for *Strongyle* and *Nematodirus* nematode resistance, respectively. Pickering et al. [25] observed an estimate of 0.25 in New Zealand sheep, and more recently, Benavides et al. [55] reported a heritability estimate of 0.36 in Australian Merino flock. Riley et al. [56] found a low heritability estimate for FAM in a Merino flock in South Africa, with values ranging from 0.06 to 0.24. The heritability estimate obtained for FAM pointed out that genetic progress for this trait is feasible, and this trait should be included in Santa Inês breeding programs. Moreover, the FAM has low cost and it is easily measured. In general, all traits showed genetic variability, therefore, it is important to investigate the presence of genomic regions or genes affecting these traits so as to elucidate and better understand their genetic architecture, especially for *Haemonchus contortus*, since it is one of the most predominant, highly pathogenic and economically important gastrointestinal parasite in sheep 5.

### GWAS, genomic regions and enrichment analysis

**Fig. 3:**
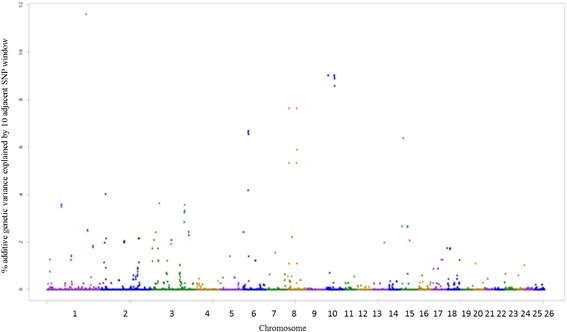
Manhattan plot of the additive genetic variance explained by windows of 10 adjacent SNPs for hematocrit (HCT) trait

**Fig. 4:**
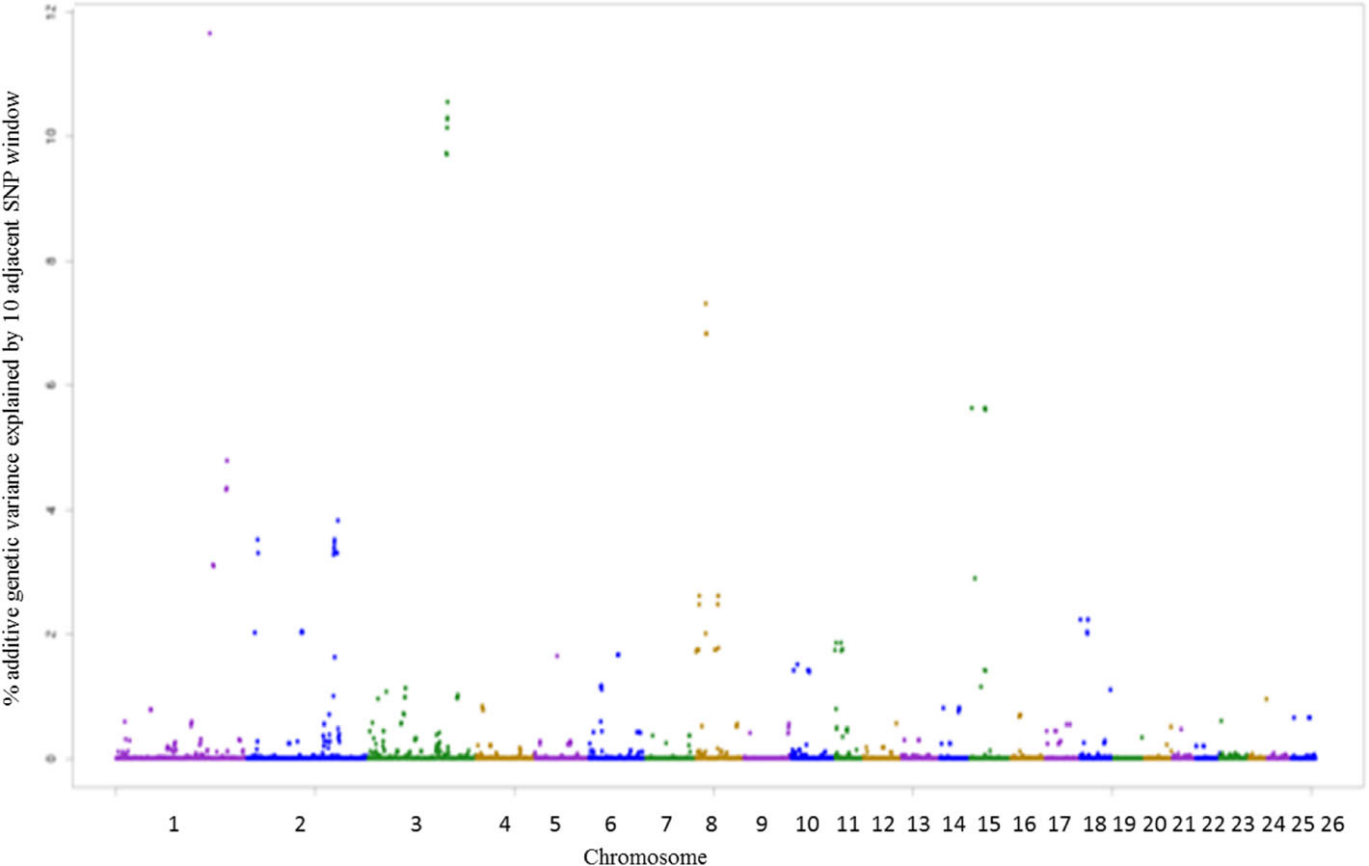
Manhattan plot of the additive genetic variance explained by windows of 10 adjacent SNPs for hemoglobin (HGB) trait

The SNPs windows regions which accounted for more than 1% of the genetic additive variance were used to search for candidate genes (CG), which are described in Additional file 1. A total of 22, 21, 23, 20, 26, 25 and 23 windows for EPG_log_, FAM, WBC, RBC, PLT, HCT and HGB traits were identified, respectively. Among the associated windows, 10 are common for HCT and HGB traits, located on OAR1, OAR2, OAR3, OAR5, OAR8 and OAR15 (Additional file 1: Table S9 and S10 – Figs. 3 and 4).

**Fig. 5:**
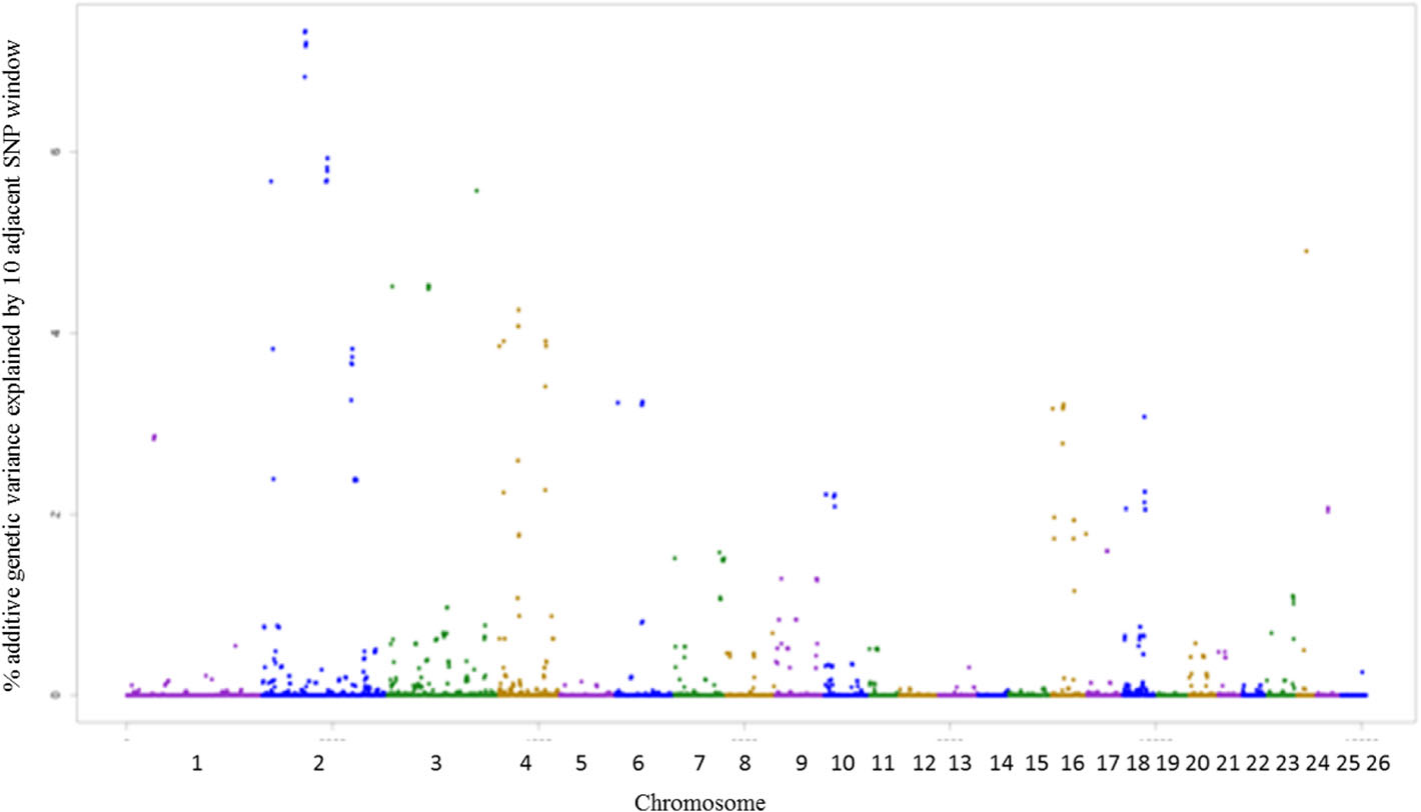
Manhattan plot of the additive genetic variance explained by windows of 10 adjacent SNPs for egg count per gram of feces (EPG_log_) trait

**Fig. 6:**
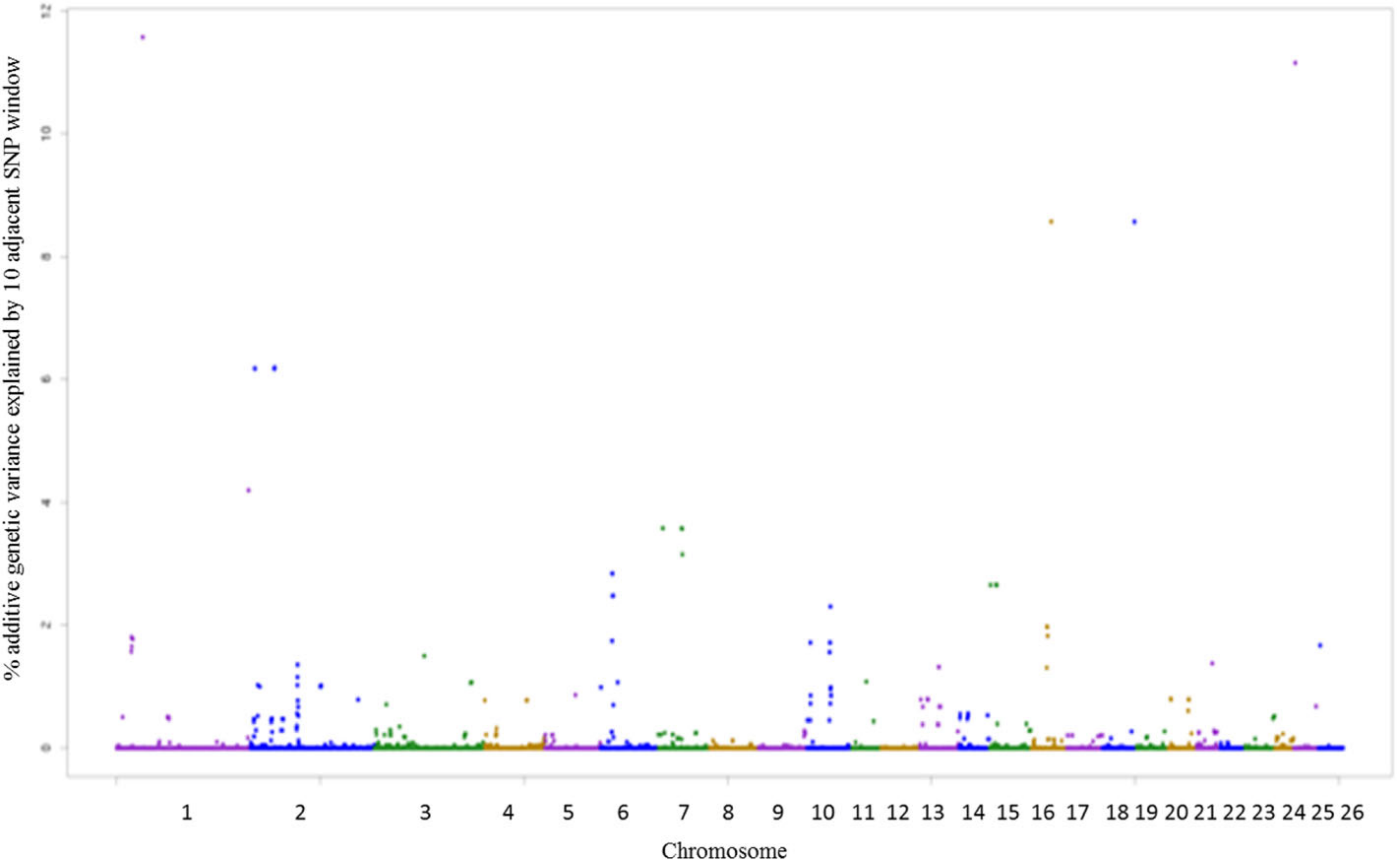
Manhattan plot of the additive genetic variance explained by windows of 10 adjacent SNPs for famacha (FAM) trait

The genomic regions associated with EPG_log_ are detailed in Additional file 1: Table S4 and illustrated in Fig. 5. Genes such as *CYP11A1* and *CYP1A1* located on chromosome 18, and *CYP19* and *SFXN1* located on chromosome 7 have functions associated with transportation or construction of iron molecules, and its absence in the blood can be an indicator of anemia in animals. The *B2M*, *SFXN1*, *IL25*, *BMP4*, *TSHR*, *CCL28*, *PIK3R1*, *FGF10*, *IL15*, *IL2*, *TP-1*, *BPMG*, *BCL10*, *HSPD1*, and *MALT1* genes are described to have functions related to the body’s immune and defense response. Indeed, these genes participate in metabolic pathways related to the development of the immune system and in the regulation of the immune process effects. Atlija et al. [26] also observed that the *IL25* gene is involved in EPG trait in adult sheep, with functions also linked to immune response. The enrichment analysis revealed the PI3K-Ark signaling pathway, which controls several cellular responses and has important functions in the immune system by regulating many key events in the inflammatory response related to damage and infection [57]. The genomic regions associated with FAM are described in Additional file 1: Table S5 and illustrated in Fig. 6. The identified CGs have functions related to the development of the immune system and the body’s defense response. Genes, such as *ADAM10*, *IL6ST*, *TNFRF13B*, *SIVA1*, *JUN*, *PAX1*, *PIK3R1*, *SIT1*, and *AKT1* are related to the differentiation of T cells, a group of white blood cells (leukocytes) responsible for defending the body against antigens. The *GUCY1A2* gene, which interacts selectively and non-covalently with iron Fe and the *SIVA1* gene, both are expressed in different subpopulations of T and B cells and provide co-stimulatory signals for the proliferation of T and B cells and immunoglobulin production by B cells [58]. The *GUCY1A2* gene operates in the process of regulating the proliferation and elimination of T cells, maintaining its number stable in the absence of external stimulus.

The results of the enriched genes and functional grouping analyses showed that genes associated with FAM are involved in functions related to body’s immune response. The biological processes (Additional file 2 – Table S11 and S12) showed that these genes are significantly (*P* < 0.05) involved in lymphocytes and leukocytes homeostasis.

**Fig. 7:**
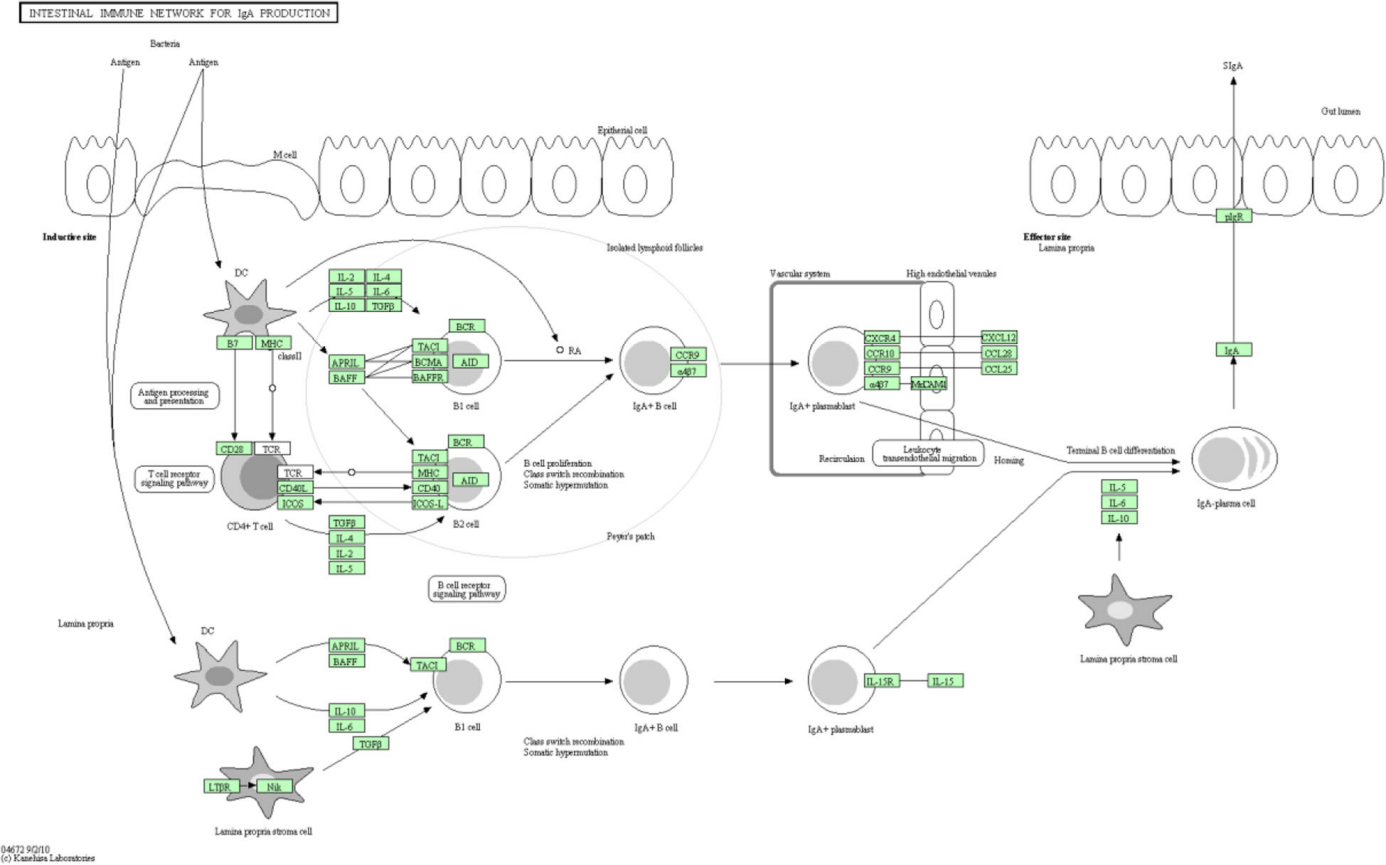
Metabolic pathway involving genes present for egg count per gram of feces (EPG) and famacha (FAM) traits

The *CCL28* gene located on OAR16 identified both for EPG and FAM acts as a chemotactic for CD4 and CD8 inactive T cells [59]. The metabolic pathways associated with the *CCL28* gene and with others associated with EPG_log_ and FAM traits, whose functions were found related to immunoglobulin synthesis in the intestine, are presented in Fig. 7.

**Fig. 8:**
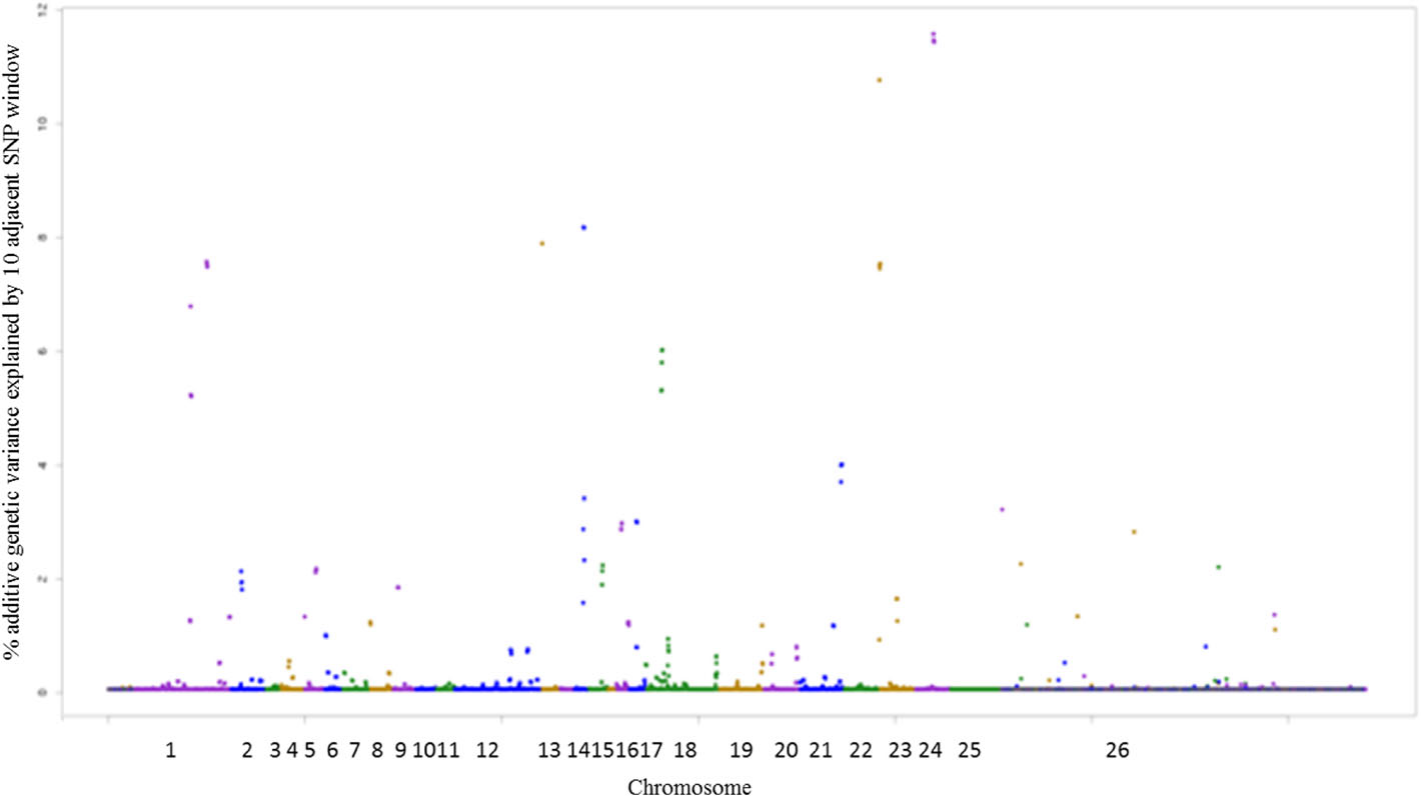
Manhattan plot of the additive genetic variance explained by windows of 10 adjacent SNPs for white blood cells (WBC) trait

The genomic regions associated with WBC are described in Additional file 1: Table S6 and illustrated in Fig. 8. The genes found in the SNP windows encode proteins that are involved in many immune system processes, responding to any potential internal or invasive threat. The vertebrate’s immune system is composed by the innate immune system (defense responses mediated by germline encoded components that directly recognize components of potential pathogens) and by the adaptive immune system which consists of T- and B-lymphocytes [60]. Many different innate immune mechanisms are deployed for host defense, a unifying theme of innate immunity is the use of germline-encoded pattern recognition receptors for pathogens or damaged self-components, such as the toll-like receptors, nucleotide-binding domain leucine-rich repeat (LRR)-containing receptors, retinoic acid-inducible gene I-like RNA helicases and C-type lectin receptors [61], like the CRCP (CGRP receptor component (CRCP)).

Some of these genes (Additional file 1: Table S6) have already been documented in others species and been associated with immune response. Beside the functions described above, they also present some particularities, i.e. the *EPBH1* (EPH Receptor B1), which is involved in the GPCR pathway and developmental biology. The first process involves the G protein–coupled receptors (GPCRs) that constitute a large protein family of receptors that recognize molecules outside the cell and activate inside signal transduction pathways and, finally, cellular responses [62].

The *GATA3* (GATA binding protein 3) gene also acts on the defense response, reacting to the presence of a foreign body or to the occurrence of an injury, restricting the injury/damage extension or preventing/recovering it from the infection caused by the foreign body. The *GATA3* gene also positively regulates T cell differentiation and any process that activates or increases the frequency, rate or extent of T cell differentiation. An additional gene having a peculiar function is the *SAMHD1* (SAM domain and HD domain 1), which is also associated with the regulation of the innate immune responses. The *SLA2* (Src-like-adaptor 2) gene acts in the T cell regulation and negative regulation of B cell activation, and in any process that stops, prevents, or reduces the frequency, rate or extent of B cell activation.

The genes in the enrichment pathways (Additional file 2), such as the *MYPN* (myopalladin), *PDGFRL* (platelet derived growth factor receptor like), *ROR1* (receptor tyrosine kinase-like orphan receptor 1), *CNTN1* (Contactin 1) and *FANK1* (fibronectin type 3, and ankyrin repeat domains 1) are associated with the Immunoglobulin-like domains that are related in both sequence and structure, and can be found in several diverse protein families. Ig-like domains are involved in a variety of functions, including cell-cell recognition, cell-surface receptors, muscle structure and the immune system [63].

Additional file 1: Table S7 and S8 show the genes with functions associated with immune response, innate, and adaptative immune response or those participating in the regulation of innate immune response for the genomic regions associated with RBC and PLT traits, respectively

**Fig. 9:**
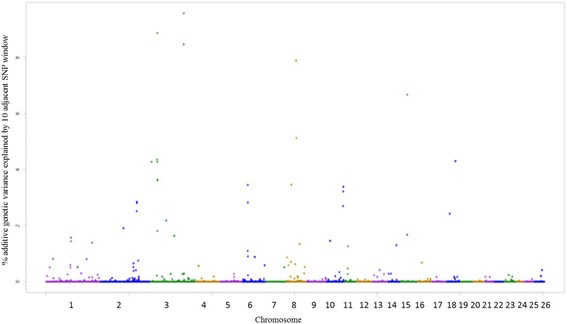
Manhattan plot of the additive genetic variance explained by windows of 10 adjacent SNPs for red blood cells (RBC) trait

The genomic regions and genes associated with RBC are presented in Additional file 1: Table S7 and illustrated in Fig. 9. The KEGG pathays analysis revealed PI3K-Ark and toll-like receptor signaling pathways significantly enriched for WBC trait (Additional File 2). The *CD109* gene (CD109 antigen) is a GPI-linked cell surface antigen expressed by CD34+ acute myeloid leukemia cell lines, T-cell lines, activated T lymphoblasts, endothelial cells, and activated platelets [64]. This gene was also reported by [26], indicating that *CD109* gene potentially contributes to resistance to gastrointestinal nematodes in sheep.

The *IL2RB* gene (interleukin 2 receptor), which is involved in T cell-mediated immune responses, is present in three forms with respect to ability to bind interleukin 2. The low affinity form is a monomer of the alpha subunit and it is not involved in signal transduction. The intermediate affinity form consists of an alpha/beta subunit heterodimer, while the high affinity form consists of an alpha/beta/gamma subunit heterotrimer. Both the intermediate and high affinity forms of the receptor are involved in receptor-mediated endocytosis and transduction of mitogenic signals from interleukin 2 [provided by RefSeq, Jul 2008]. Kondo et al. [65] showed that a clonogenic common lymphoid progenitor, a bone marrow-resident cell that gives rise exclusively to lymphocytes (T, B, and natural killer (NK) cells), can be redirected to the myeloid lineage by stimulation through exogenously expressed interleukin (IL)-2 and GM-CSF (granulocyte/macrophage colony-stimulating factor) receptors. As the *IL2RB* is one of the receptors that act in IL-15 expression, it has been proposed to be a critical cytokine for NK cell development. This gene can affect this redirections in cytokine signaling and can regulate cell-fate decisions. Additionally, a critical step in lymphoid commitment is downregulation of cytokine receptors that drive myeloid cell development.

Poliovirus receptor-like proteins (PVRLs), such as PVRL4, are adhesion receptors of the immunoglobulin superfamily and function in cell-cell adhesion [66]. The encoded protein contains two immunoglobulin-like (Ig-like) C2-type domains and one Ig-like V-type domain. It is involved in cell adhesion through trans-homophilic and -heterophilic interactions. It is a single-pass type I membrane protein [provided by RefSeq, Jan 2011].

The *CXCR4* gene may influence the immune system under physiologic and pathologic conditions through negative regulation of MHC class II expression, possibly through PKA and SRC kinase [67]. In a study, Contento et al. [68] observed that while human T cell activation by antigen-presenting cells is taking place, the *CCR5* and *CXCR4* chemokine receptors are recruited into the immunological synapse, where they deliver costimulatory signals. This gene also participates in the intestinal immune network for IgA production pathway.

The *ASCC3, CRLF2RB, FNDC3A, FNDC4, IFNAR1, IFNAR2, LEPR, MYLK, NCAM2, PVRL4 SLAMF1, TXNIP, CD109, ACAN, NTRK3, CCDC141, LEPR, MYLK, PVLR4, IL12RB2, IGSF10*, and *JAM2* genes (Additional file 1: Table S7) are domains with an Ig-like fold that can be found in several proteins in addition to immunoglobulin molecules. For example, Ig-like domains occur in several different types of receptors (such as various T-cell antigen receptors), several cell adhesion molecules, MHC class I and II antigens, as well as the hemolymph protein hemolin, and the muscle proteins titin, telokin and twitchin (IPR013783).

**Fig. 10:**
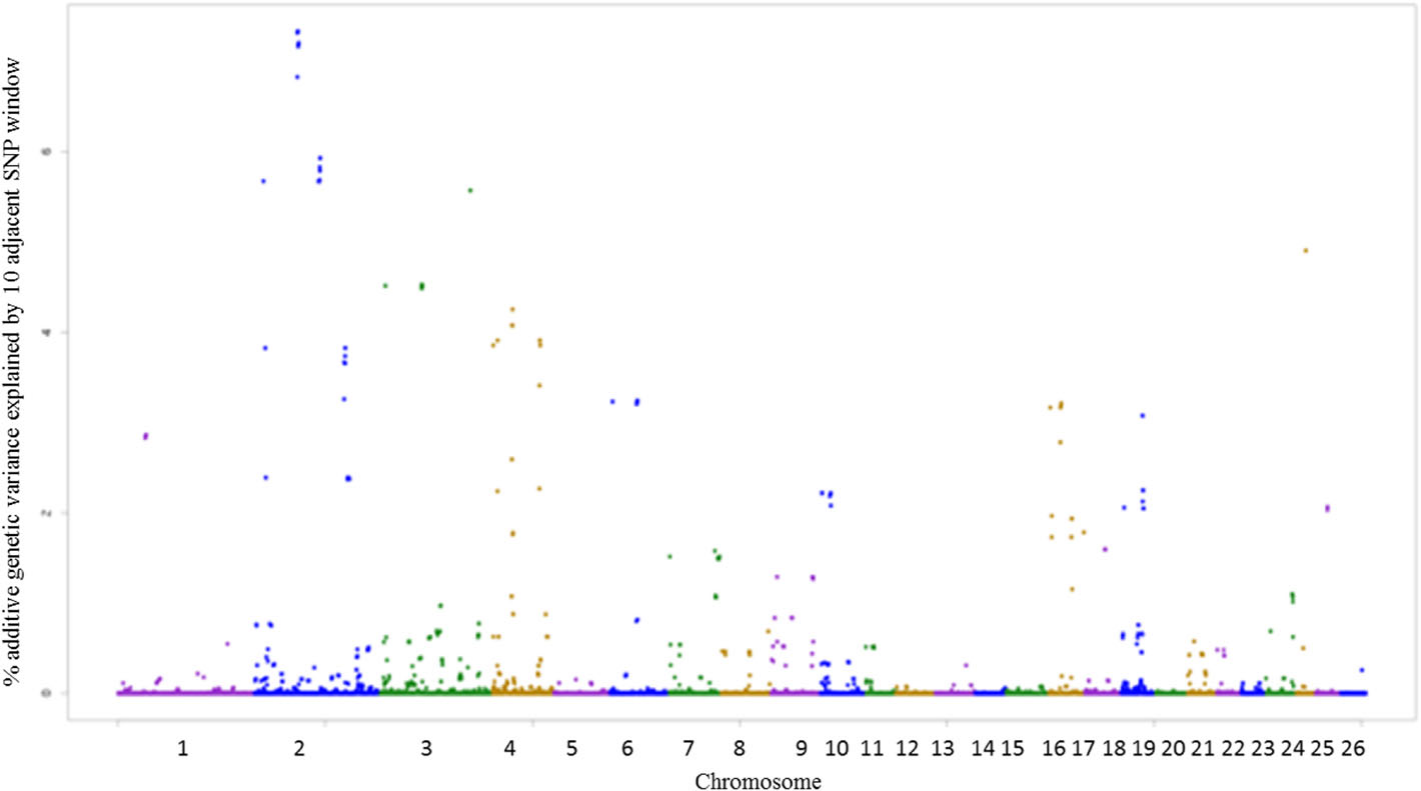
Manhattan plot of the additive genetic variance explained by windows of 10 adjacent SNPs for platelet (PLT) trait

The KEGG pathway analysis revealed platelet activation, toll-like receptor and PI3K-Ark signaling pathways for PLT (Additional file 2). The *CXCL1, CXCL8* and *CXCL10* genes identified for PLT (Additional file 1: Table S8; Fig. 10) belong to a subfamily of chemokines that basically are structurally related to molecules that regulate cell trafficking of various types of leukocytes through interactions with a subset of 7-transmembrane and G protein-coupled receptors. They also play a fundamental role in the development, homeostasis, and functionality of the immune system [69].

Different genes were also identified for PLT, in these regard, the Fc-gamma receptors (FCGRs), such as *FCGR1A, FCGR3A*, and *FCGR2B*, that are integral membrane glycoproteins which exhibit complex activation or inhibitory effects on cell functions after aggregation by complexed immunoglobulin G (IgG) [70]. These genes encode proteins that play an important role in the immune response, a receptor for the Fc portion of immunoglobulin G, and participate in the removal of antigen-antibody complexes from the circulation, as well as other antibody-dependent responses. It is also involved in the phagocytosis of immune complexes and in the regulation of antibody production by B-cells, respectively [provided by RefSeq, Jul 2008; RefSeq, Jun 2010].

Atlija et al. [26] observed some significant chromosome-wise QTL detected by linkage analysis and from the combined LD and linkage analysis. These authors reported some CG involved in immune response. Theirs findings support our results, since genes such as *MBN, BTC, CXCL1, CXCL10, EREG, RASSF6, SCARB2, TMPRSS11D, AMBN,* and *AREG* were observed in both studies, indicating that these genes can be used and deply studied as CG for resistance to gastrointestinal nematodes in sheep. Some of the genes cited in Additional file 1: Table S9 and S10 have functions related to immune response or to immunoglobulin development, and were associated with HCT and HBG traits, respectively.

For HCT trait (Table S9), genes like *IGF1R, IFNAR1, IFNAR2, IL12RB2, IL2RB, C2F2RB, PVRL4, LEPR, MYLK, SLAMF1, ADGRF3*, and *TXNIP* were identified. These genes encode domains with an Ig-like fold that can be found in several proteins in addition to immunoglobulin molecules. For example, Ig-like domains occur in several different types of receptors (such as T-cell antigen receptors), many cell adhesion molecules, MHC class I and II antigens, as well as the hemolymph protein hemolin, and the muscle proteins titin, telokin and twitchin.

The *IL1A* and *IL1B* genes have the protein encoded as a member of the interleukin 1 cytokine family. This cytokine is a pleiotropic cytokine involved in several immune responses, inflammatory processes, and hematopoiesis [provided by RefSeq, Jul 2008]. Also the *IL1B* gene, initially discovered as the major endogenous pyrogen, induces prostaglandin synthesis, neutrophil influx and activation, T- and B-cells activation, cytokine production, antibody production, fibroblast proliferation and collagen production. Also it was observed a function related to ligand-binding domain that displays similarity to C2-set immunoglobulin domains (antibody constant domain 2) in the *LEPR* gene. It has a specific effect on T lymphocyte responses, differentially regulating the proliferation of naive and memory T-cells.

For HCT and HGB traits, genes related to immune response were identified, i.e. the *RAC2* gene, that seems to increase the resistance to parasites. In order to understand the function of RAC2 GTPase in regulating the cellular immune response, Williams et al. [71] assayed the effects of hemocytes in parasitized *RAC2* mutant larvae, as well as characterized the effect of over-expressing *RAC2* gene in hemocytes. These authors reported that this gene has an important role in the cellular immune response, being necessary for hemocyte spreading and cell-cell contact formation during immune surveillance against the parasitoid *L. boulardi*. When an invading parasitoid is recognized as a foreign body, circulating hemocytes should recognize them and attach to the egg chorion. After the attachment, the hemocytes should then spread out to form a multilayered capsule surrounding the invader. In *RAC2* mutants this process was disrupted. Besides of that, this gene also acts on the B cell receptor signaling pathway, like the *PPP3CA* gene.

The *HSPD1* gene encodes a mitochondrial protein that plays a role as a signaling molecule in the innate immune system. Zanin-Zhorov et al. [72] reported that this gene, as well as a synthetic peptide derived from it, acted as a costimulatory of human regulatory CD4-positive/CD25 (*IL2RA*) positive T cells (Tregs), which inhibit lympho proliferation and IFNG and TNF secretion by CD4-positive and CD8-positive T cells. The authors concluded that the self-molecule *HSPD1* can downregulate adaptive immune responses by upregulating Tregs through *TLR2* signaling.

The *CD80* and *CD86* genes identified in almost all traits (RBC, PLT and HCT) probably participate effectively in the activation of T cells, which requires engagement of two separate T-cell receptors. The antigen-specific T-cell receptor (TCR) binds foreign peptide antigen-MHC complexes, and the CD28 receptor binds to the B7 (*CD80*/C*D86*) costimulatory molecules expressed on the surface of antigen-presenting cells (APC). The simultaneous triggering of these T-cell surface receptors with their specific ligands results in the activation of this cell. Many in vitro and in vivo studies demonstrated that both CD80 and CD86 ligands have an identical role in the activation of T cells. Recently, functions of B7 costimulatory molecules in vivo have been investigated in B7-1 and/or B7-2 knockout mice, and the authors concluded that *CD86* could be more important for initiating T-cell responses, while *CD80* could be more significant for maintaining these immune responses [73]. Recently, [24] observed some important genes matching to important pathways involved in host immune response against parasites. The *PDGFRA* gene (OAR6: 67,950,121–69,892,816) was also observed by Benavides et al. [24], whose findings have pointed out as a key gene in cytokine signaling.

## Conclusions

The traits indicating gastrointestinal parasites resistance shown adequate genetic variability to respond to selection in Santa Inês breed, and it is expected a higher genetic gain for famacha when compared to the others traits. The level of LD estimated for markers separated by less than 1 Mb indicates that the Ovine SNP12k BeadChip will likely be a suitable tool for identifying genomic regions associated with those traits related to gastrointestinal parasite resistance.

Several candidate regions related to immune system development and activation, inflammatory response, regulation of lymphocytes, and leukocytes proliferation were found in this study. These candidate regions and CG may help in the selection of animals with higher resistance to parasites, and consequently, reduce the anthelmintic drugs costs, also the production losses linked to the use of it. Furthermore, when reducing the use of anthelmintic drugs, a potential reduction in waste problems regarding meat and milk discharge due to drugs residues should be minimized, reducing the impact upon the environment.

## Additional files

Additional file 1: Table S4. Genomic regions associated with egg counts per gram of feces (EPG_log_) in Santa Inês sheep; **Table S5.** Genomic regions associated with famacha (FAM) in Santa Inês sheep; **Table S6.** Genomic regions associated with blood cell count (WBC) in Santa Inês sheep; **Table S7.** Genomic regions associated with red blood cells (RBC) in Santa Inês sheep; **Table S8.** Genomic regions associated with platelet (PLT) in Santa Inês sheep; **Table S9.** Genomic regions associated with hematocrit (HCT) in Santa Inês sheep; **Table S10.** Genomic regions associated with blood cell count (HGB) in Santa Inês sheep. (XLS 74 kb)

Additional file 2: Table S11. KEGG pathways (*P* < 0.05) for counting eggs per gram of feces (EPG_log_), famacha (FAM), white blood cells (WBC), red blood cells (RBC), platelets (PLT), hematocrit (HCT), and hemoglobin (HGB) traits obtained from the DAVID software; **Table S12.** Gene Ontology terms for biological processes significantly (*P* < 0.01) related with counting eggs per gram of feces (EPG_log_), famacha (FAM), white blood cells (WBC), red blood cells (RBC), platelets (PLT), and hematocrit (HCT) traits. (XLS 68 kb)

## Abbreviations

BCS: Body condition score
EPG_log_: Egg counts per gram of feces
FAM: Degree of anemia assessed by the famacha card
FC: Feces consistency
FS: Fur score
HCT: Hematocrit
HGB: Hemoglobin
LD: Linkage disequilibrium
PLT: Platelets
RBC: Red blood cell count
WBC: White blood cell count
